# Evaluation of cerebral blood flow change after cigarette smoking using quantitative MRA

**DOI:** 10.1371/journal.pone.0184551

**Published:** 2017-09-27

**Authors:** Yunsun Song, Joong-goo Kim, Hong-Jun Cho, Jae Kyun Kim, Dae Chul Suh

**Affiliations:** 1 Departments of Radiology and Research Institute of Radiology, University of Ulsan, College of Medicine, Asan Medical Center, Seoul, Korea; 2 Department of Family Medicine, University of Ulsan, College of Medicine, Asan Medical Center, Seoul, Korea; 3 Department of Radiology, Chung-Ang University, College of Medicine, Seoul, Korea; Medical Photonics Research Center, Hamamatsu University School of Medicine, JAPAN

## Abstract

**Background and objective:**

Changes in cerebral blood flow (CBF) immediately after cigarette smoking (CS) are still unclear. Our purpose was to evaluate the hemodynamic changes in each intracranial vessel after CS by using quantitative magnetic resonance angiography (MRA).

**Material and methods:**

Fifteen healthy male smokers less than 45 years old with more than 3 pack-year smoking history were enrolled in this study. The hemodynamic change in the vessels, represented by cerebral flow rate (CFR, ml/s) and flow velocity (FV, cm/s), was quantitatively measured in eleven vascular segments of the brain using phase-contrast MRA. Two sets of data at each vessel before and after CS were statistically analyzed by paired t-test. Three of 15 participants, as a control group, followed all the procedures but did not smoke.

**Results:**

Total CFR of the distal intracranial vessels (anterior, middle, and posterior cerebral arteries; ACA, MCA, and PCA) was significantly reduced after CS by 7.3% (847 vs. 785 ml/s, *p* = 0.024). Such flow changes were statistically more significant in the anterior circulation (ACA and MCA) compared to the posterior circulation (PCA). All distal intracranial vessels did not have significant FV change while peak systolic velocity and mean velocity dropped 7.4 and 4.3% and pulsatility index decreased 10.9% in the internal carotid artery. Regarding cross-sectional areas, all distal intracranial vessels showed diminished, and only MCA had a statistical significance (9.9 vs. 9.3 mm^2^, *p* = 0.016).

**Conclusions:**

There was a significant decrease of CFR after CS especially in the anterior circulation of twelve young male smokers. Considering the changes of FV and cross-sectional area all together, it can be suggested that cerebrovascular impedance increased after CS especially at the main trunk level of the distal intracranial vessels (ACA, MCA, and PCA).

## Introduction

Cigarette smoking (CS) is an important risk factor for cerebral atherosclerosis [1–3]. The smoke is a toxic aerosol that includes more than thousands of chemicals such as nicotine, carbon monoxide, acrolein, and oxidant material [4–8]. Cumulative harmful effects of CS may contribute to leukocyte telomere attrition and the pathogenesis of chronic diseases such as atherosclerosis [9–11]. In the United States at present, about two-thirds of ever smokers start smoking before 20 years of age [12]. This trend is also observed in other countries [13]. Although the CS rate in Korea has dropped over the last 20 years, 21.6% of Koreans over 20 years of age still smoke, with the male smoking rates (male: female = 37.6 vs. 5.8%) being the second highest among OECD (Organisation for Economic Co-operation and Development) countries in 2014 [14]. Many Korean adolescents spend, on average, 2 hours a day in computer gaming rooms, which increases their exposure to smoke fumes and their desire to smoke [15].

The effect of CS on the brain has been discussed in many studies [16–31]. Cerebral blood flow (CBF) is defined as the volume of blood passing through a given amount of brain tissue per unit of time (ml/100g/min). Alteration of the CBF after CS is a particularly important issue, as it may affect functional aspects of the brain and be related to smoking addiction. In many studies with various modalities, including xenon inhalation, positron emission tomography (PET), and single-photon emission tomography, the reported effects of CS on CBF vary considerably depending on the region of the brain and among the studies [16, 21–24, 26–28, 30]. Correlations with arterial nicotine level on regional CBF were statistically significant with certain brain areas such as cerebellum and occipital cortex having the greatest increases in relative blood flow [26, 27]. In these studies, CBF was estimated from the distribution of administrated radioactive material rather than direct evaluation of the flow in the vessel itself, which could be the fundamental cause of the CBF change.

A recent Transcranial Doppler (TCD) study revealed that flow velocity (FV) increased to a different degree in all distal intracranial vessels (anterior, middle, and posterior cerebral arteries; ACA, MCA, and PCA) after smoking one cigarette [29]. Because the pulsatility index (PI) decreased after CS, the study concluded that the direct effect of CS on cerebral circulation included possible constriction of the proximal trunk of the intracranial arteries with peripheral vasodilation. However, there are some limitations of TCD device itself when assessing the cerebral flow rate (CFR). Since TCD can evaluate only the FV of a vessel, the exact flow amount through the vessel would not be quantified. Obtaining appropriate sonic window for evaluating intracranial vessels is also challenging.

We summarized the prior literature regarding the effect of CS on the brain (Table 1). A summary of the flow-related terminology, study tools, and target anatomical areas used in the previous studies is presented in Table 2. Variable changes of the CBF and FV after CS were observed depending on the study and cerebral region. These may reflect the complex mechanisms of the effects of CS on the brain as well as the limitations of current imaging modalities. On the premise that certain hemodynamic effects of CS may exist, we formulated hypotheses that the underlying mechanism of the cerebral hemodynamic change perhaps lies on the cerebral blood vessels, and each vessel may exhibit a different response to CS. Thus, to investigate the response of each intracranial vessel and subsequent CBF modification after CS, we performed exact localization and subsequent quantitative flow measurements of intracranial vascular segments by using TOF MRA and phase-contrast MR.

**Table 1:** Preveious cerebral blood flow studies on cigarette smoking.

Authors	Participants		Modality	Results	
	Smokers	Non-smokers		Increased	Decreased
Yamamoto et al.[24] (2003)	10	5	SPET (^99m^Tc-ECD)	-	Whole brain-CBF
Rose et al.[32] (2003)			PET (H_2_^15^O)	Left frontal region-CBF	Left amygdala-CBF
Domino et al.[26] (2004)	19		PET (H_2_^15^O)	Occipital cortex, cerebellum, thalamus-CBF	Anterior cingulate, nucleus accumbens, amygdala, hippocampus-CBF
Zubieta et al.[27] (2005)	17		PET (H_2_^15^O)	Visual cortex, cerebellum-CBF	Anterior cingulate, right hippocampus, ventral striatium-CBF
Shinohara et al.[28] (2006)	10		PET (H_2_^15^O)	-	-
Cruickshank et al.[17] (1989)	6		Xenon/TCD	MCA-FV	Whole brain-CBF
Morioka et. al.[33] (1997)	4		TCD	ICA,VA-FV	**-**
Kodaira et al.[18] (1993)	25		TCD	MCA-FV	MCA-PI
Silvestrini et al.[19] (1996)	24	24	TCD	MCA-FV	**-**
Boyajian et al.[20] (2000)	14		TCD	MCA-FV	**-**
Barutcu et al.[25] (2004)		16	TCD	CCA-FV	CCA-PI
Kochanowicz et al.[29] (2015)	36		TCD	ACA,MCA,PCA-FV	ACA,MCA,PCA-PI

SPET, single-photon emission tomography; ^99m^Tc-ECD, technetium-99m-labelled ethylcysteinate dimer; PET, Positron emission tomography; H_2_^15^O, ^15^O-labeled water; FV, flow velocity; CBF, cerebral blood flow; PI, pulsatility index; ACAs, bilateral anterior cerebral arteries; MCAs, bilateral middle cerebral arteries; PCAs, bilateral posterior cerebral arteries; VAs, bilateral vertebral arteries; ICAs, bilateral internal carotid arteries.

**Table 2:** Summary of flow-related terminology, study tool and target anatomical area.

Flow parameter	Study tool	Target anatomical area
Cerebral Blood Flow (CBF)	PET, Xenon, SPECT	Brain tissue
Flow velocity (FV)	DUS	Intra- and extra-cranial vessel
	Phase-contrast MRA (NOVA)	Intra- and extra-cranial vessel
Blood flow volume	DUS	Extra-cranial vessel
Cerebral Flow Rate (CFR)	Phase-contrast MRA (NOVA)	Intra- and extra-cranial vessel

Abbreviation: DUS, Doppler Ultrasound; MRA, Magnetic resonance angiography; PET, Positron emission tomography; SPECT, Single photon emission computed tomography.

Quantitative magnetic resonance angiography (Q-MRA) has been used to assess cerebral hemodynamics for identifying high risk patients for stroke and guiding treatment decisions [34]. This is based on the two-dimensional (2D) phase-contrast magnetic resonance angiography (PC-MRA) by which blood flows can be measured in intracranial vessels in a simple, noninvasive manner and without contrast agent. This MR technique is established on the principle that magnetic field gradients introduce a phase shift in flowing spins that is proportional to blood flow velocity[35]. On the basis of this phase shift, hemodynamic parameters of FV and cross-sectional area can be directly measured for each vessel from which CFR can be calculated. Therefore, we expect to obtain more accurate and objective information regarding the hemodynamic change and its mechanism as well. Our purpose was to evaluate the hemodynamic changes in each intracranial vessel after CS by using Q-MRA.

## Materials and methods

### Study population and setting

Fifteen healthy male volunteers (24–45 years, mean 32.3) who are currently smokers were recruited by advertisement (3–15 pack-year, mean 7.0). Volunteers more than 45 years old or female were excluded to minimize any possible effect of atherosclerosis or hormonal influence. Participants were screened for a history of heart or lung disease, stroke, hepatic or renal disease, malignancy, hypertension, diabetes, and hypercholesterolemia, and were excluded if they had any of above illnesses. Subjects who were taking any medications were also excluded. This study was reviewed and approved by the Asan Medical Center Institutional Review Board (2015–0735). The subjects provided written informed consent.

Subjects were instructed to cease CS at least 8 hours ahead of the study, and we assured their compliance with an expiratory test using a carbon monoxide (CO) detector. Expired air CO level exceeding 10 ppm was considered as non-compliance, and the subject required thorough review to confirm sufficient smoking abstinence.

When the subjects entered the MRI suite, their initial heart rate (HR) and blood pressure (BP) were measured while they were seated. After baseline MRI, which included Time-of-Flight (TOF) sequence and quantitative flow analysis, were taken, the participants went outside and smoked one cigarette in 5 minutes. The cigarettes given to the participants were of the same brand and contained 0.7 mg of nicotine and 8 mg of tar. The distance between the MRI suite and the smoking place was about 100 meters. It took about 3 minutes by slow walk. After CS, the subjects’ BP, HR, and MRI were measured again under the same conditions.

Basically, the design of this study is within-subject comparisons between pre-, and post-smoking status. Three of 15 participants, who were control group, followed all procedures and received both MR scans but did not smoke.

### MRI acquisition and quantitative analysis

All the participants underwent cardiac-gated PC-MRI (TR, 7.2 to 8.1 milliseconds; TE, 4.6 to 5.3 milliseconds; flip angle, 15°; slice thickness, 5 mm for ICAs and 4 mm for other arteries; FOV, 160 mm for ICAs and 140 mm for other arteries; matrix, 192×96 for ICAs and 192×144 for other arteries) on a 3 Tesla Philips scanner (MR systems Achieva release 3.2.2.0).

**Fig 1:**
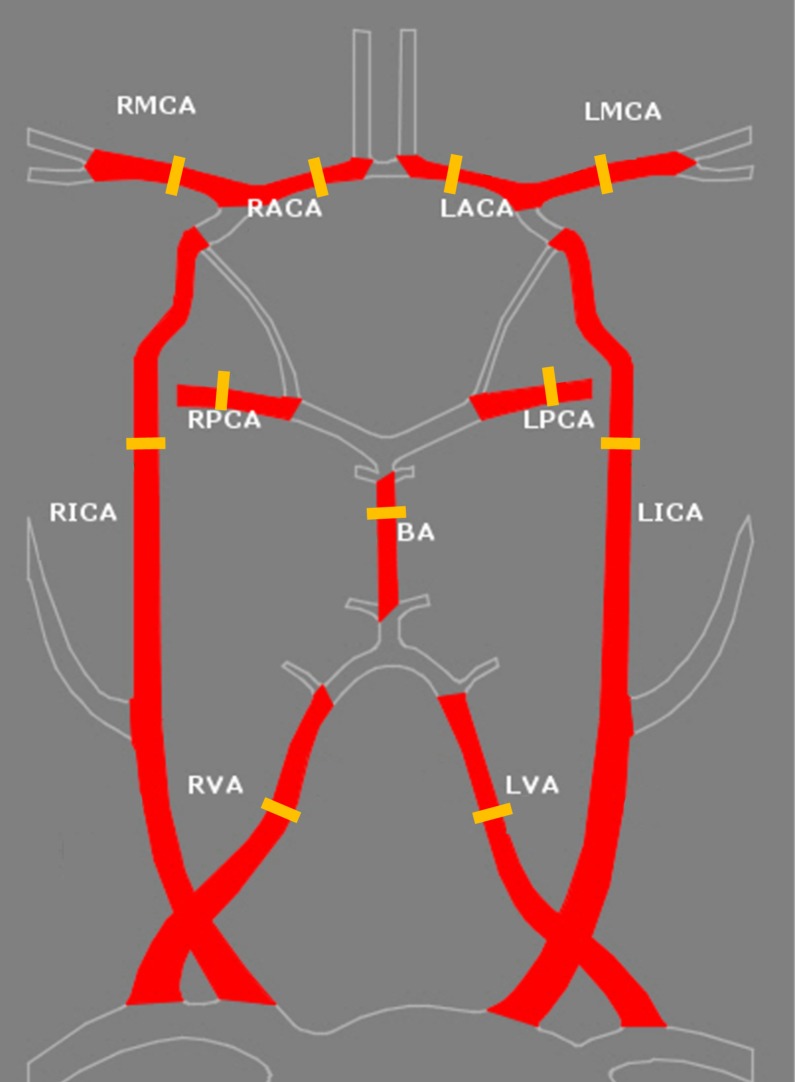
Location of flow measurements in the intracranial and extracranial vessels. Both sides of the anterior cerebral arteries (ACAs), the middle cerebral arteries (MCAs), the posterior cerebral arteries (PCAs), the internal carotid arteries (ICAs), the vertebral arteries (VAs), and the basilar trunk (total of 11 vessels).

**Fig 2:**
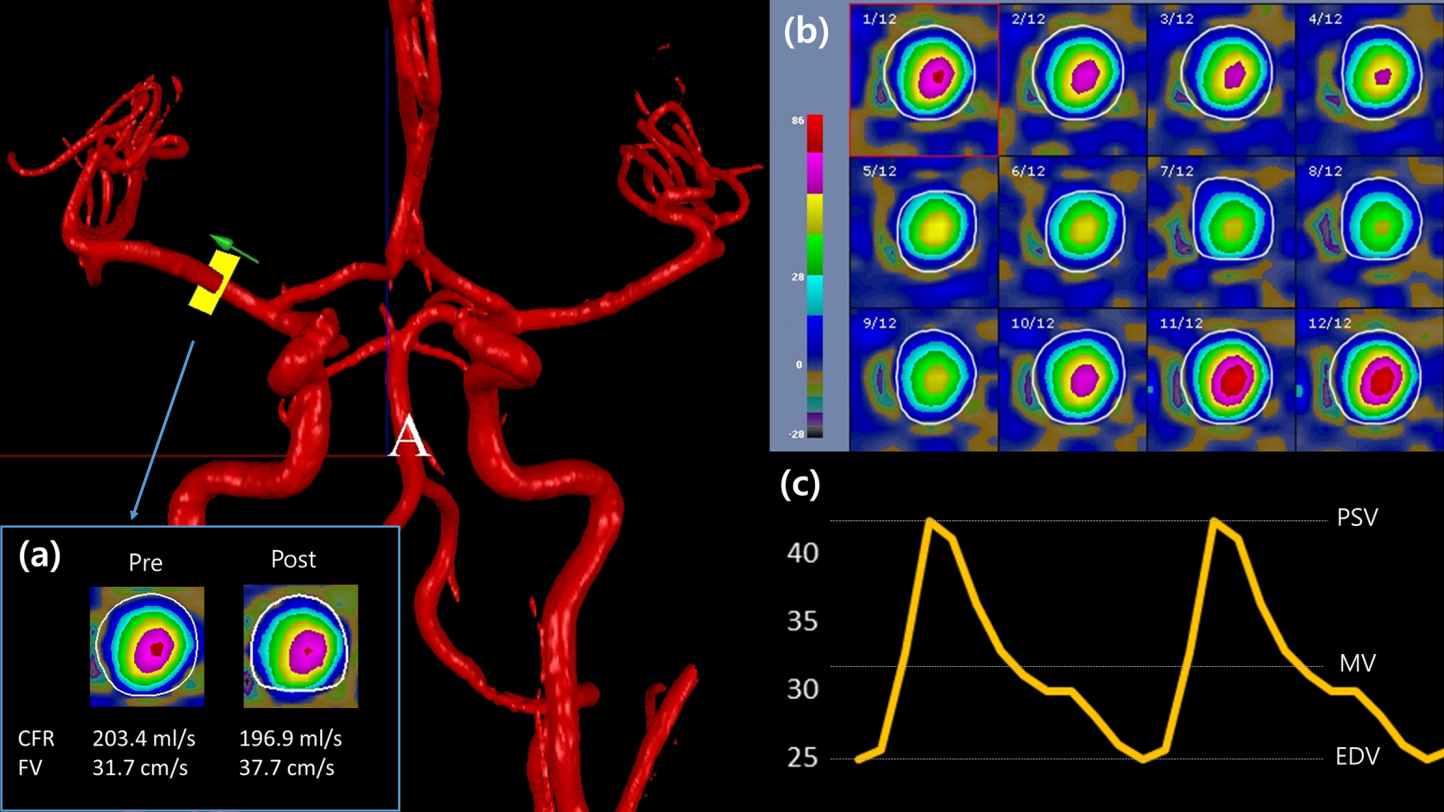
An example of verification process of NOVA for velocity encoding in the target vessel (flow analysis in right MCA, case 10). (a) Velocity maps of the M1 segment of the right MCA before and after cigarette smoking (CS) shows decreased flow rate and increased flow velocity after CS. (b) Velocity maps according to the phases of one cardiac cycle (pre-smoking) verify that there is no aliasing for high velocities within the velocity range chosen for phase encoding. (c) Flow velocity curve based on the above velocity maps. FV (flow velocity) in (a) represents MV (mean velocity) in (c). PSV and EDV were only used for calculating pulsatility index (PI). CFR indicates cerebral flow rate; FV, flow velocity; PSV, peak systolic velocity; MV, mean velocity; EDV, end-diastolic velocity.

Noninvasive Optimal Vessel Analysis (NOVA) software (VasSol, Chicago, IL, USA) was used for the quantitative cerebral flow measurement. Prior to the flow measurement, TOF images were obtained for the 3D modeling of the blood vessels. Then, the standard point for the quantitative measurement in the eleven vessels were set; cervical segments of the internal carotid arteries (ICAs), intradural (V4) segments of the vertebral arteries (VAs) below the posterior inferior cerebellar artery (PICA) origin, distal basilar trunk below the superior cerebellar artery (SCA) origin, A1 segments of both ACAs, M1 trunk of the MCAs, and P2 segments of the PCAs (Fig 1). Using the rotational 3D navigation tools, the operator placed an imaginary plane perpendicular to the centerline of the target vessel. The position was pre-specified for consistency between pre- and post-smoking localizations, and the location was selected at the furthest distance from any branching or curved vessels within the target segment to minimize potential effects of turbulence. The contour of the vessels at the selected location was automatically demarcated on the velocity maps (Fig 2). Manual contouring was required in some cases by separating the vessel margin when the vessel contour margin overlapped with adjacent vessels. As doing so, average cross-sectional area (mm^2^) can be obtained. The FV through the specific point of the vessel was quantitatively measured by using 2D PC-MRA. Finally, CFR was calculated from the area and FV by NOVA software.

The results of hemodynamic analysis were provided with the values of CFR (ml/s), flow velocity (cm/s), and cross-section area of vessel (mm^2^) at each cardiac phase moment which was variable depending on the HR of the participants (9~13 phases per second). PI values were calculated in each vessel by subtracting the end-diastolic velocity (EDV) from the peak systolic velocity (PSV) and then dividing by the mean velocity (MV) (Fig 2).

### Statistical analysis

**Fig 3:**
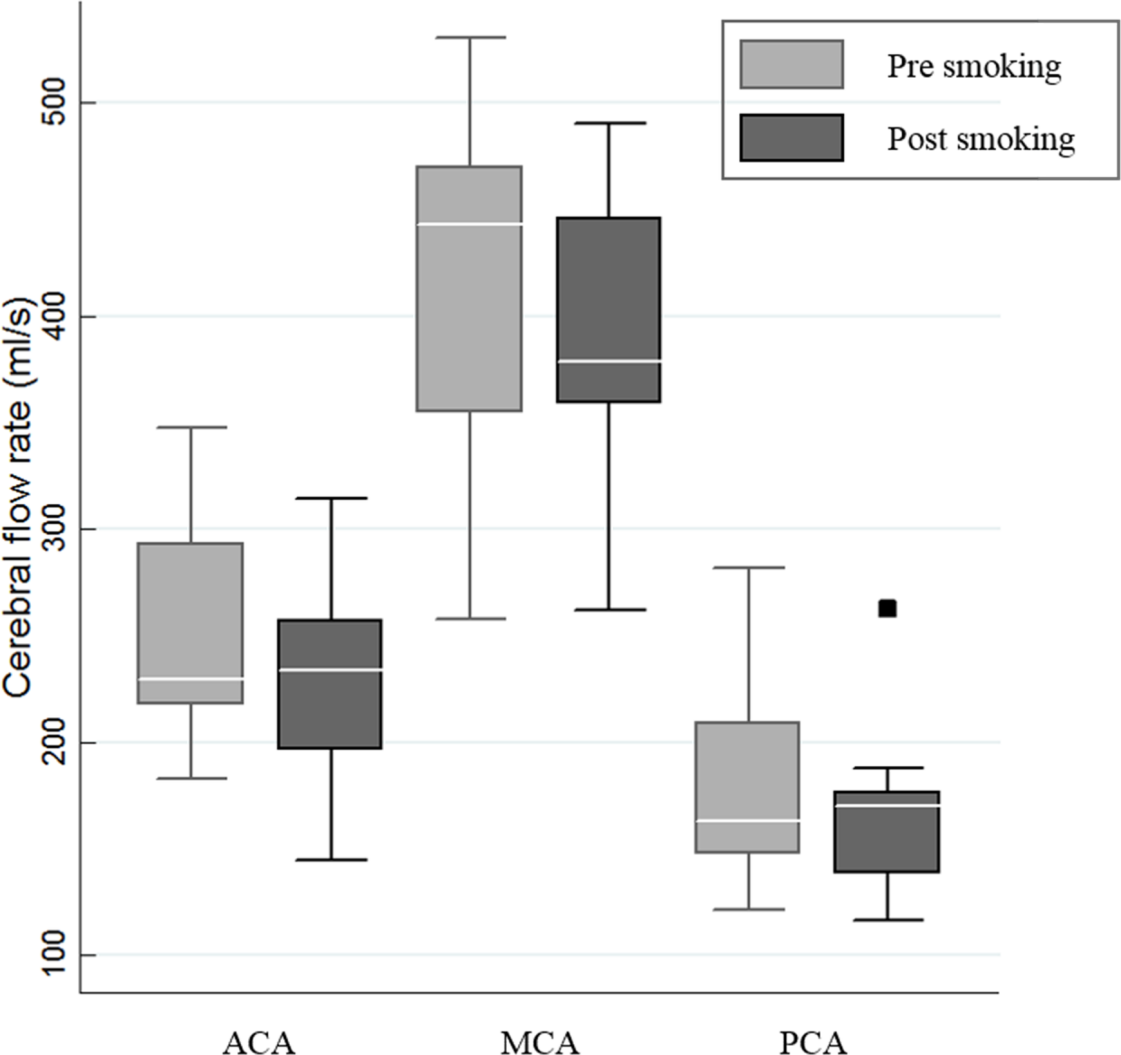
Cerebral flow rate changes in intracranial vessels after cigarette smoking (CS). CFR (ml/s) in the anterior cerebral artery (ACA), the middle cerebral artery (MCA), and the posterior cerebral artery (PCA) before and after CS was demonstrated using box plot. Box = 25^th^, 75^th^ percentiles; black bars = minimum and maximum values; white bar = median value; square = outlier values.

Continuous variables were expressed as mean ± standard deviation. Paired t-test was used to estimate the significance of differences in blood flow parameters before and after CS. For some parameters that could not be assumed to be normally distributed, Wilcoxon signed-rank test was used instead. Shapiro-Wilk test was used for normality evaluation. A probability of less than 0.05 indicated statistical significance. In Fig 3, a box plot demonstrates the CFR changes in distal intracranial vessels after CS. All statistics were computed using STATA version 13.0 (StataCorp LP, College Station, TX).

## Results

The participants were comprised of smoking group (*n* = 12) and non-smoking group as a control (*n* = 3). Baseline characteristics were demonstrated on Table 3. There was no significant difference in age and smoking history between the groups. Baseline mean BP and HR also did not show any difference. In smoking group, however, mean BP and HR significantly increased after CS while non-smoking group did not show significant change.

**Table 3:** Baseline characteristics.

	Smoking (n = 12)		Non-smoking (n = 3)		
		*p-value		*p-value	^#^p-value
Age (years)	32.0 ± 1.5	-	33.6 ± 5.7	-	0.63
Pack-year	6.8 ± 3.7	-	8.0 ± 7.5	-	0.69
Pre mBP (mmHg)	96.7 ± 11.6	0.02	97.4 ± 3.6	0.5	0.91
Post mBP (mmHg)	104.5 ± 11.6		91.4 ± 13.1		
Pre HR (bpm)	65.1 ± 10.2	0.002	66.6 ± 6.6	0.08	0.81
Post HR (bpm)	73.6 ± 13.2		56.6 ±1.6		

All parameters are expressed as mean ± SD. mBP, mean blood pressure; HR, heart rate; bpm, beat per minute.

*Paired T-test was used to compare between pre and post-smoking.

^**#**^Paired T-test was used to compare between smoking and non-smoking group.

When it comes to CBF changes after CS, there was no significant difference between both sides of the vessel segments on the flow parameters; therefore, the sum of the values of both sides was used for analysis. The CFR changes of the different vessels after CS are demonstrated in Table 4. One patient did not have the information of the ACA because of technical problem (probably due to excessive vascular motion during cardiac phases). The CFR of the distal intracranial vessels (ACA, MCA, and PCA) was significantly reduced after CS by 7.3% (847 vs. 785 ml/s, *p* = 0.024). Both anterior (ACA and MCA) and posterior (PCA) circulations decreased in CFR by 7.5% and 6.8% respectively, however, only anterior circulation showed significant reduction (669 vs. 619 ml/s, *p* = 0.015). In the anterior circulation, CFRs of both ACA (250 vs. 230 ml/s, *p* = 0.018) and MCA (419 vs. 389 ml/s, *p* = 0.031) were significantly dropped although ACA showed a slightly larger change than MCA (-8.2 vs. -7.0%) (Fig 3). On the other hand, proximal vessels including cervical segment of ICA and intradural VA demonstrated subtle variable changes without statistical significance.

**Table 4:** Change of cerebral flow rate after cigarette smoking (N = 12 smokers).

Artery (N = 12)	Cerebral flow rate (ml/s)		Difference (%)	P-value *
	Before smoking	After smoking		
Intracranial total (N = 11) (ACAs + MCAs + PCAs)	847 ± 157	785 ± 140	-7.3	0.024
Anterior circulation (N = 11) (ACAs + MCAs)	669 ± 125	619 ± 113	-7.5	0.015
ACAs (N = 11)	250 ± 52	230 ± 51	-8.2	0.018
MCAs	419 ± 83	389 ± 70	-7.0	0.031
PCAs	178 ± 44	166 ± 38	-6.8	0.17
Cervical ICAs	618 ± 113	617 ± 125	-0.3	0.93
Intradural VAs	260 ± 69	265 ± 62	2.0	0.34
Basilar artery	160 ± 51	161 ± 45	0.3	0.95

All parameters are expressed as mean ± SD. ACAs, bilateral anterior cerebral arteries; MCAs, bilateral middle cerebral arteries; PCAs, bilateral posterior cerebral arteries; VAs, bilateral vertebral arteries; ICAs bilateral internal carotid arteries.

*Paired T-test was used.

The changes of flow velocity and cross-sectional area of the vessels are demonstrated in Table 5. Most of the velocity parameters of ICA except EDV changed significantly. PSV and MV dropped 7.4 and 4.3% each and PI decreased 10.9%. VA also had significantly lower PI value. All distal intracranial vessels did not have significant velocity alteration while PI values of the MCA and PCA declined more than 10% despite it was not significant. Regarding cross-sectional areas, all distal intracranial vessels showed diminished while only MCA had a statistical significance (9.9 vs. 9.3 mm^2^, *p* = 0.016). The areas of proximal vessels including ICA and VA slightly increased without significance after CS.

**Table 5:** The changes of cerebral blood flow velocity and cross-sectional area after cigarette smoking.

	Before smoking	After smoking	Difference (%)	P-value
**ACA (n = 12)**				
PSV (cm/s)	33.3 ± 5.8	34 ± 5.6	2.1	0.48
MV (cm/s)	27.7 ± 5.1	28.1 ± 4.7	1.5	0.54
EDV (cm/s)	23 ± 4.7	22.9 ± 4.5	-0.1	0.97
PI	0.38 ± 0.09	0.4 ± 0.08	5.3	0.43
Area (mm^2^)	7.4 ± 1.4	6.9 ± 1.2	-6.7	0.057
**MCA (n = 12)**				
PSV (cm/s)	45.4 ± 8.9	43.6 ± 9.4	-3.9	0.29
MV (cm/s)	36 ± 7.2	35.9 ± 8.4	-0.4	0.91
EDV (cm/s)	28.5 ± 5.9	29.2 ± 8.1	2.6	0.51
PI	0.47 ± 0.09	0.41 ± 0.11	-12.3	0.07
Area (mm^2^)	9.9 ± 2.3	9.3 ± 2.1	-5.9	**0.016**
**PCA (n = 12)**				
PSV (cm/s)	31.3 ± 4.2	31 ± 4.6	-1.0	0.74
MV (cm/s)	25.3 ± 3.5	25.7 ± 3.9	1.6	0.55
EDV (cm/s)	19.7 ± 3.4	21.1 ± 3.4	7.2	0.054
PI	0.46 ± 0.06	0.39 ± 0.11	-16.2	0.11
Area (mm^2^)	5.9 ± 1.2	5.4 ± 0.9	-8.1	0.10
**ICA (n = 12)**				
PSV (cm/s)	33.7 ± 6.9	31.2 ± 6.2	-7.4	0.006^*^
MV (cm/s)	24.9 ± 4.1	23.8 ± 4.4	-4.3	0.018^*^
EDV (cm/s)	18.4 ± 3.1	18.3 ± 3.7	-0.6	0.81
PI	0.61 ± 0.09	0.54 ± 0.12	-10.9	**0.030**
Area (mm^2^)	20.8 ± 3.5	21.8 ± 4	4.6	0.12
**VA (n = 12)**				
PSV (cm/s)	23.2 ± 4	22.6 ± 3.9	-2.7	0.49
MV (cm/s)	17.6 ± 3.1	17.6 ± 3.3	0.1	0.98
EDV (cm/s)	13 ± 2.7	13.5 ± 2.9	4.1	0.35
PI	0.59 ± 0.1	0.52 ± 0.09	-11.1	**0.011**
Area (mm^2^)	12.2 ± 2.5	12.5 ± 2.2	2.2	0.58*

*Wilcoxon signed-rank test was used; for the other variables, paired T-test was used. All parameters are expressed as mean ± SD. PSV, peak systolic velocity; MV, mean velocity; EDV, end-diastolic velocity; PI, pulsatility index; ACA, the anterior cerebral artery; MCA, the middle cerebral artery; PCA, the posterior cerebral artery; ICA, cervical segment of the internal carotid artery; VA, the vertebral artery.

The non-smoking control group (*n* = 3) did not show any significant findings regarding CFR, flow velocity, and cross-sectional area of the vessels (S1 Table).

## Discussion

Our study showed that CFR (ml/s) in all the distal intracranial vessels (ACA, MCA, and PCA) demonstrated decreasing trends after CS, while only the anterior circulation (ACA and MCA) was confirmed to have significant changes, unlike the posterior circulation. Flow velocity did not show significant change after CS although PI of distal intracranial vessels demonstrated decreasing trends without significance. Considering that CFR = FV x Cross-sectional area of a vessel wall, reduction of CFR meant decreased cross-sectional area without change of FV. “Cross-sectional area” in our study decreased 6.7% (p = 0.057) and 5.9% (p = 0.016) respectively which had at least marginal significance. Another noticeable thing we found is decreased cross-sectional area of the distal vessels (5.9 ~ 8.1%) suggesting vasoconstriction even though only MCA was statistically significant.

Previous TCD studies had to depend on the PI and Lindegaard index[36] to understand complex intracranial hemodynamics (i.e. peripheral vascular impedence, MCA vasospasm). However, without measuring cross-sectional area, flow rates of the vessels cannot be completely estimated. Most distinguishable point of the present study is an advantage of direct measuring CFR from the flow velocity during a cardiac cycle at the cross-sectional vessel area by using quantitative flow analysis with PC-MRA.

A recent study using TCD revealed that the flow velocity of all intracranial arteries increased to a different degree after CS: The end-diastolic velocity increased more significantly than the PSV, and thus, showed decrease of PI suggesting flow-mediated vessel dilatation [18, 29, 37]. These results led to the conclusion that smoking reduces vascular resistance in cerebral arteries and increases CBF. Other previous studies using TCD also have a consistent trend of increased flow velocity of the MCA (Table 5).

**Fig 4:**
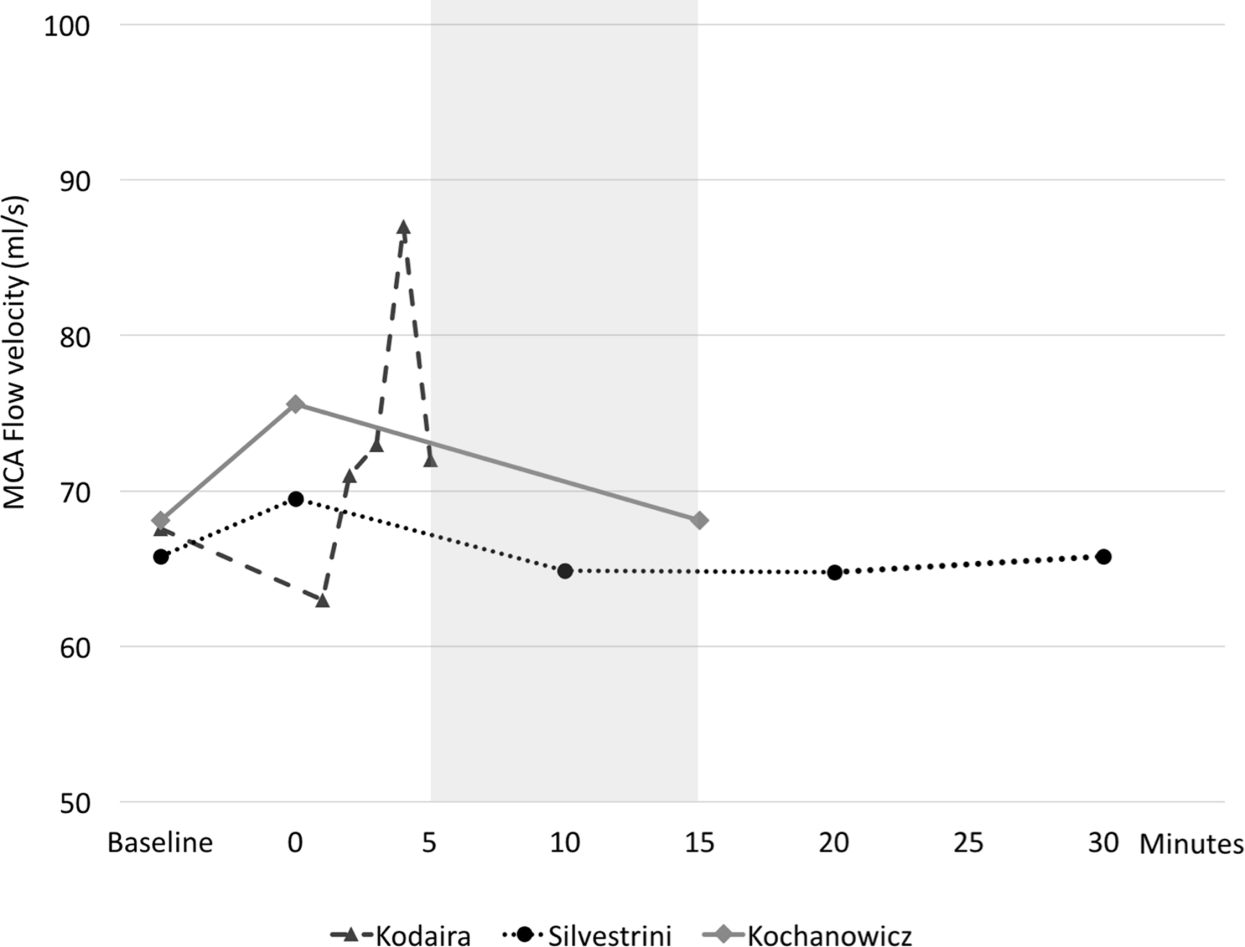
The sequential changes of the middle cerebral artery (MCA) flow velocity before and after cigarette smoking (CS) in previous literatures[18, 19, 29]. The graph shows MCA flow velocity changes over time. The ‘baseline’ means before CS, and the ‘0 minutes’ indicates the point immediately after CS. The gray area between 5 and 15 minutes is the period in which we performed quantitative measurement in this study. The velocity changes during the time period of our study corresponds to decreasing trend of velocity described in two other reports[19, 29].

A possible reason why the current study did not reveal increased flow velocity after CS could be the time gap between the CS and the MR study (average 10 minutes in total: 3 minutes to return to the MR suite after CS; 2 minutes to undergo TOF MRI scan; 5 minutes for quantitative flow analysis). We compared the time gap observed in our study with those of three other studies that mentioned the exact time point of the measurement after CS (Fig 4). According to the flow velocity change observed in the study by Kodaira et al., the CS effect on the MCA flow velocity seemed to gradually increase in the early period and then abruptly dropped after 4 minutes [18]. Kochanowicz et al. also showed that the blood flow parameters in the major cerebral arteries returned to the pre-smoking level within 15 min [29]. These results may reflect the short-acting effect of CS, and the effect on the flow velocity in our study could have disappeared during the time gap. However, the CFR change noticed in our study might persist longer than the flow velocity change.

Some findings which are concordant to the aforementioned TCD studies are increased EDV and declined PI of the vessels. Increased flow velocity, especially in the diastolic component of TCD, was regarded as reduced peripheral vascular resistance [18, 29]. Based on decreased CFR in our study, increased diastolic component in the large intracranial cerebral vessel may not necessarily represent increased cerebral perfusion. It could be explained that there was stronger effect of the vasoconstriction of main trunk in the distal arteries (ACA, MCA, and PCA) than the possible compensatory effect of the vasodilation of peripheral branches.

Both BP and HR increased significantly after CS. The fact that non-smoking group did not show significant change and even decreasing average BP and HR confirmed that there was no or minimal effect of physical activity walking to the smoking place on the BP and HR.

It is uncertain why smoking affects more anterior intracranial vessels. A possible explanation is that the toxic effects of smoking preferentially affect the intracranial vessels of the anterior circulation because antioxidant or enzymes that protect from oxidative stress are more abundant in the intracranial arteries than in extracranial arteries [38, 39]. Such preferential local effects may also lead to proportionally more common intracranial stenosis in younger age groups with relatively less exposure to other risk factors found in older people, such as hypertension and diabetes [40–42]. In addition, risk factors such as diabetes and hypertension are relatively more closely related to posterior circulation atherosclerosis [43].

There were several limitations in this study. First, only one measurement of Q-MRA at the certain time point was obtained after CS because our MR protocol took certain times to get TOF MRA and obtain flow information from the eleven vascular segments. Therefore, the study did not show instant changes in time sequence. Further study tracing only one vascular segment and continuous monitoring every minute could be optimal to see the timely longitudinal changes after CS. Second, smoking effect may vary according to the smoking history of each study individual. The one who has less exposure to smoking may have been affected more by the smoking compared to the other who has longer and heavier smoking history. Third, flow velocity and CFR in each vascular segment may not be interchangeably interpreted in the same individual because the data from each anatomical segment of cerebral arteries may have reciprocal relationship through the Circle of Willis or the leptomeningeal collaterals. Finally, although we designed this study as a within-subject comparison model in which we compared the values between pre- and post-smoking of a group, the negative control group was rather small. We could not completely exclude possibility of hemodynamic change in normal controls (negative control) which underwent the same study without smoking. The control group (n = 3, number of vessels = 33) showed no significant hemodynamic change after all the same process except smoking. Even though the control group (n = 3, number of vessels = 33) showed no significant hemodynamic changes, we could not completely exclude the possibility of hemodynamic change in normal controls (negative control) due to its small sample size. Further study with large sample or simplification of the smoking processes may be required.

In summary, exact localization and subsequent quantitative flow measurement of intracranial vascular segments by using TOF MRA and phase-contrast MR in our study revealed that CFR was significantly decreased after CS. The degree of the CFR reduction was more dominant in the anterior circulation than in the posterior circulation. Our study showing decreased CFR after CS suggested increased cerebrovascular impedance especially at the main trunk level of the distal intracranial vessels (ACA, MCA, and PCA).

## Supporting information

S1 Table Change of cerebral flow rate after cigarette smoking (N = 3, control group). (DOCX)
