# Supplementary material for: Evaluation of cerebral blood flow change after cigarette smoking using quantitative MRA
Source: PLoS One. 2017 Sep 27;12(9):e0184551. doi: 10.1371/journal.pone.0184551 (PMC5617327; doi:10.1371/journal.pone.0184551)
Supplement: S1 Table — (DOCX) [file pone.0184551.s001.docx]

**S1 Table. Change of Cerebral Flow Rate after Cigarette Smoking (N=3, control group)**

| **Artery (N=3)** | **Cerebral flow rate** (ml/s) | | **Difference (%)** | **P-value** ^*^ |
| --- | --- | --- | --- | --- |
|  | **Before smoking** | **After smoking** |  |  |
| Intracranial total  (ACAs + MCAs + PCAs) | 743.8 ± 105.5 | 812.8 ± 108.6 | 9.3 | 0.10 |
| Anterior circulation  (ACAs + MCAs) | 582.3 ± 101.4 | 632.3 ± 90.5 | 8.6 | 0.10 |
| ACAs | 220 ± 43.6 | 249.2 ± 28.6 | 13.3 | 0.10 |
| MCAs | 362.3 ± 65.8 | 383 ± 71.2 | 5.7 | 0.10 |
| PCAs | 161.6 ± 9.5 | 180.6 ± 24 | 11.8 | 0.10 |
| Cervical ICAs | 587.1 ± 50.1 | 596.3 ± 69.7 | 1.6 | 0.59 |
| Intradural VAs | 268.5 ± 29.5 | 260.8 ± 18.1 | -2.8 | 0.59 |
| Basilar artery | 181.2 ± 22.8 | 186.6 ± 24.4 | 3.0 | 1.0 |

^*^Wilcoxon signed-rank test was used
